# Incidence of fracture hospitalization and surgery during pregnancy in Finland—1998–2017: a retrospective register-based cohort study

**DOI:** 10.1007/s00402-023-04931-w

**Published:** 2023-06-13

**Authors:** Lauri Nyrhi, Ilari Kuitunen, Ville Ponkilainen, Tuomas T. Huttunen, Ville M. Mattila

**Affiliations:** 1 Department of Surgery, Central Finland Hospital Nova, Jyväskylä, Finland; 2 Faculty of Medicine and Health Technology, Tampere University, Tampere, Finland; 3 Department of Pediatrics, Kuopio University Hospital, Kuopio, Finland; 4 School of Medicine, University of Eastern Finland, Kuopio, Finland; 5 Heart Center, Tampere University Hospital, Tampere, Finland; 6 Department of Musculoskeletal Surgery, Tampere University Hospital, Tampere, Finland

**Keywords:** Pregnancy, Trauma, Fracture, Epidemiology, Incidence

## Abstract

**Introduction:**

The aim of this study was to assess the incidence of all major fractures and surgery during pregnancy and the outcomes of pregnancy in Finland between 1998 and 2017.

**Materials and methods:**

A retrospective cohort study using nationwide data from the Finnish Care Register for Health Care and the Finnish Medical Birth Register. As participants we included all women aged between 15 and 49 years from January 1, 1998 to December 31, 2017 and their ≥ 22-week pregnancies.

**Results:**

Of a total 629,911 pregnancies, 1813 pregnant women were hospitalized with a fracture diagnosis, yielding an incidence of 247 fractures/100,000 pregnancy-years. Of these, 24% (*n* = 513/2098) were treated operatively. The most common fractures were fractures of the tibia, ankle, and the forearm, which made up half of all fractures. The incidence of pelvic fractures was 6.8/100,000 pregnancy-years, with an operation rate of 14%. The stillbirth rate of all fracture patients was low at 0.6% (*n* = 10/1813), although this was 1.5-fold the overall stillbirth rate in Finland. Lumbosacral and comminuted spinopelvic fractures resulted in preterm delivery in 25% (*n* = 5/20) of parturients, with a stillbirth rate of 10% (*n* = 2/20).

**Conclusion:**

The incidence of fracture hospitalization during pregnancy is lower than in the general population, and fractures in this population are more often treated conservatively. A higher proportion of preterm deliveries and stillbirths occurred in women with lumbosacral and comminuted spinopelvic fractures. Maternal mortality and stillbirth rates remain low among women with fractures leading to hospitalization or surgery during pregnancy.

**Supplementary Information:**

The online version contains supplementary material available at 10.1007/s00402-023-04931-w.

## Introduction

Traumatic injury is the leading non-obstetrical cause of maternal and fetal death during pregnancy [1]. The exact incidence of traumatic injuries during pregnancy is not known, but it has been estimated to affect approximately 8% of pregnancies [2]. In addition to increasing maternal and perinatal mortality, trauma has also been reported to increase the risk for preterm birth, cesarean section, premature membrane rupture, and placental abruption [3–5]. A previous case study showed that traumatic injury of the pelvis or acetabulum during pregnancy can lead to pregnancy termination in 35–60% of cases [6]. In patients with polytrauma, (ISS[7] > 12) fetal mortality has been evaluated to be 65% [8]. The most common injury mechanisms during pregnancy are collisions between motor vehicles and pedestrians, and falls [9].

A small number of pregnant women in their third trimester suffer from transient pregnancy-associated osteoporosis, which has been associated with low-energy spinal column fractures and delivery-related proximal femur fractures [10]. Transient osteoporosis during pregnancy has previously been hypothesized to be underdiagnosed as a cause of back pain during pregnancy [11].

To date, no large-scale epidemiological studies investigating the incidence of fractures leading to hospitalization during pregnancy have been published. However, whole-population incidences of pelvic and acetabular fractures, some of the most critical fractures regarding pregnancy and delivery, have recently been reported to be 73 and 11 per 100,000 person-years, respectively [12]. Moreover, the number of pregnant women treated operatively for pelvic and acetabular fractures has been reported to be 4 times fewer than females in the general population [12]. Operative treatment methods for fractures during pregnancy vary, and even the most invasive locking-plate fixations and intramedullary nails are not excluded during pregnancy [13, 14].

The aim of our study is to analyze all fractures leading to hospitalization among pregnant women and the normal population of fertile-aged women in Finland between 1998 and 2017 to provide exact data on the incidences of fractures leading to hospitalization and subsequent operations. As a secondary outcome, we aim to report the outcome rates of pregnancies influenced by severe trauma.

## Materials and methods

Data for this nationwide retrospective register-based cohort study were obtained from the Finnish Health and Social Data Permit Authority (FinData). We combined data from the Finnish Care Register for Health Care and the Medical Birth Register. The Finnish Care Register includes hospital inpatient data as well as data from day surgeries and specialized outpatient care. The coverage and accuracy of the registers have been proven to be excellent, although information regarding patient comorbidities is lacking [15–17]. The Medical Birth Register contains information on all pregnancies ending in delivery after gestational week 21 + 6 or fetal weight over 500 g. The validity and coverage of the register are excellent [18].

Our study period was from January 1, 1998 to December 31, 2017. Patients were selected from the Care Register using all fracture diagnoses coded with the 10th version of the International Classification of Diseases, (ICD-10) [19] and all orthopedic fracture procedure codes from the Finnish version of the Nordic Medico-Statistical Committee (NOMESCO) classification (Supplementary file 1) [20]. All female patients aged 15–49 years at the time of the injury, defined as fertile by the World Health Organization, were included in the study [21].

**Fig. 1:**
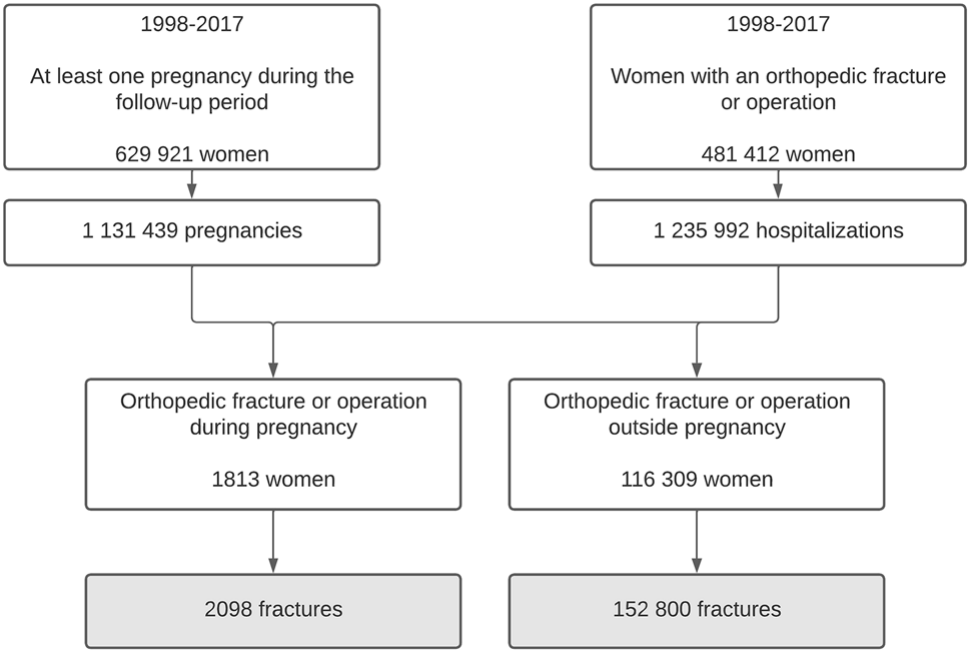
Flow chart of study cohort formation

The registers were combined after the individuals were pseudonymized by FinData. The pseudonymization key was retained by FinData and none of the authors had access to the key. Additionally, all files were analyzed by the safe remote-controlled environment provided by FinData. Using information on the date of birth and pregnancy duration from the Medical Birth Register, we were able to isolate incidents that occurred during pregnancy. In our study, the primary outcome was hospitalization with any of the fracture ICD-10 or operation codes. Only the first hospitalization per fracture or operation was taken into account. The formation of the study cohort is described in Fig. 1.

This study has been granted research permission from the Finnish Health and Social Data Permit Authority FinData, permission THL/1756/14.02.00/2020. According to Finnish research legislation and the Finnish National Board on Research Integrity appointed by the Ministry of Education and Culture, a review by a formal ethics committee is not required for the research of public and published data, registry and documentary data, and archive data [22]. Our study was formatted according to the Strengthening the Reporting of Observational Studies in Epidemiology (STROBE) guidelines for observational studies (Supplementary file 2) [23].

### Statistical analysis

Yearly incidence rates (per 100,000 pregnancy-years) were calculated for fractures and operations and divided into anatomical subgroups. Total incidences and 95% confidence intervals (CI) were then calculated for these subgroups using an estimated pregnancy length of 39 weeks. For the normal population of women of similar age, age-adjusted incidence rates (per 100,000 person-years) were calculated. Age-adjustment was conducted by first calculating the crude incidence rates for each one-year age-group of the normal population separately. These incidence rates were then weighted by multiplying the crude rate by the proportion of women in each age-group of the postpartum population and summed to yield the final age-standardized incidence rate. All incidence rates, incidence rate ratios (IRR) and 95% confidence intervals were calculated using Poisson regression. Critical fractures concerning pregnancy and patients with multiple major fractures were studied separately. As critical fractures, we included codes S32.0 (Lumbar column fracture), S32.1 (Sacral fracture), S32.3 (Iliac fracture), S32.4 (Acetabular fracture), S32.5 (Pubic fracture), S32.7 (Multiple pelvic or lumbar vertebrae fractures), S32.8 (Other pelvic fracture), S72.0 (Femoral neck fracture), S72.1 (Pertrochanteric femoral fracture), S72.2 (Subtrochanteric femoral fracture), and S72.3 (Femoral diaphyseal fracture). For the analysis of multiple major fractures, all fractures of the torso and proximal limbs were included. Patients with simultaneous multiple pelvic fractures were analyzed with code S32.7 regardless of primary coding. In addition, fracture and operation incidences with 95% CIs, preterm delivery rates, and mortality rates were calculated. In this study, trauma-related preterm delivery was defined as delivery prior to the completion of the 37th week of gestation and occurring within 7 days of trauma. This classification was decided by the authors, as no previous studies have evaluated applicable definitions. Fractures were considered surgically treated when fracture surgery was performed within 14 days of the first hospitalization. For this subgroup, only pregnancies over 22 gestation weeks were included because the Finnish Birth Register does not include earlier gestation data, as pregnancies terminated earlier are not recorded. Statistical analyses were performed using R version 4.0.3.

## Results

### All fractures

**Fig. 2:**
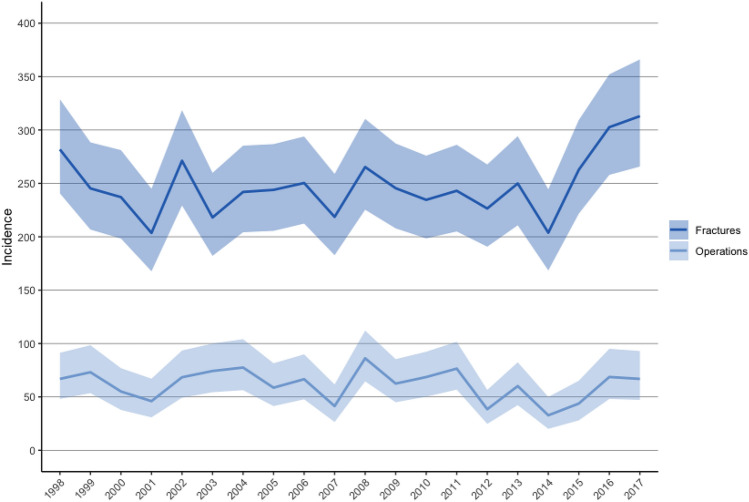
Yearly incidence (per 100,000 pregnancy-years) of fracture hospitalization and surgery during pregnancy in Finland between 1998 and 2017 and their 95% confidence intervals

The cumulative 20-year incidence for fractures was 247 per 100,000 pregnancy-years (95% CI 237–259) and 61 per 100,000 pregnancy-years (95% CI 56–67) for fracture surgery. In total, 24% (*n* = 513/2098) of the fractures underwent operative treatment. Annual incidence rates for fractures varied between 204 and 313 fractures per 100,000 pregnancy-years. Corresponding operation rates varied from 33 to 86 operations per 100,000 pregnancy-years (Fig. 2). For the normal population of women the respective 20-year incidence for all fractures was 554 per 100,000 person-years and 150 per 100,000 person-years for fracture surgery. For the normal population the total age-adjusted fracture incidence was 553 per 100,000 person-years (95% CI 536–572) for a total IRR of 0.34 (95% CI 0.33–0.34). 30% of all fractures were treated operatively in the normal population.

According to Finnish Care Register data, a total of 1813 pregnant women were hospitalized with a fracture diagnosis during the 20-year study period. In this population, a total of 2098 fractures were registered. The mean age (±SD) for all patients was 29.6 (±5.7). In-hospital maternal mortality due to trauma was low, as three mothers died as a result of trauma (incidence 0.2 per 100,000 pregnancies, 95% CI: 0–0.6). In all cases, the main cause of death was severe traumatic brain injury. The stillbirth rate was 0.9 per 100,000 pregnancies (95% CI: 0.4–1.6, 0.5% of fracture patients, *n* = 10/2098), which is 1.2-fold the stillbirth rate of the general population in Finland [18].

### Anatomical distribution of fractures

The most common anatomical location of fracture was the tibia and ankle (incidence 70.1/100,000) of which distal tibial and fibular fractures of the ankle accounted for 67% (*n* = 399/595; Table 1). Fractures of the forearm had an incidence rate of 54.9/100,000, and the rate of surgery was 16% (*n* = 76/466). Fractures of the distal radius accounted for 54% (*n* = 253/466) of all forearm fractures. The highest rate of surgical treatment was in fractures of the thigh (50%, *n* = 13/26), followed by fractures of the tibia and ankle (42%, *n* = 250/595). The incidence of pelvic fractures was 6.8 per 100,000 pregnancy-years (95% CI: 4.0–6.7), and the rate of surgical treatment was 14% (*n* = 8/59), which was the third lowest rate of surgery after fractures of the skull (0%, *n* = 0/114) and spine (6%, *n* = 6/102).

**Table 1:** Incidences of fractures and their operations divided by anatomical location

Anatomical location	Pregnant women				Normal population			
	Fractures		Surgery		Fractures		Surgery	
	Incidence (95% CI)		Incidence (95% CI)		Incidence (95% CI)		Incidence (95% CI)	
Head	13.44	(11.01–16.13)	0	(0–0.44)	41.2	(36.46–46.4)	0.40	(0.09–1.23)
Spine	12.03	(9.8–14.59)	0.82	(0.33–1.69)	27.93	(24.05–32.26)	4.01	(2.74–5.92)
Pelvis	6.83	(5.29–8.97)	0.69	(0.25–1.53)	12.53	(10.0–15.51)	1.98	(1.10–3.36)
Brachium	19.56	(16.69–22.67)	1.77	(0.99–2.92)	48.52	(43.36–54.13)	14.30	(11.63–17.42)
Forearm	54.92	(50.03–60.13)	6.72	(5.09–8.71)	120.79	(112.56–129.47)	24.25	(20.74–28.22)
Hand	33.23	(29.47–37.35)	6.24	(4.68–8.17)	84.26	(77.4–91.57)	27.94	(24.17–32.18)
Thigh	3.01	(2.0–4.49)	1.41	(0.73–2.47)	10.85	(8.49–13.67)	5.46	(3.88–7.52)
Tibia and ankle	70.12	(64.6–75.87)	25.33	(22.07–28.96)	150.81	(141.62–160.45)	64.98	(59.16–71.26)
Foot	12.37	(10.12–14.97)	3.77	(2.56–5.32)	33.70	(29.42–38.44)	15.15	(12.41–18.36)
Total	247.24	(236.77–258.05)	46.79	(42.29–51.63)	553.36	(353.56–571.60)	149.85	(140.91–159.23)

Incidences reported as fractures or operations per 100,000 pregnancy-years. Poisson exact test used to calculate 95% confidence intervals in brackets

For the normal population, all fracture all locations were more common compared to the pregnant population. Fracture incidence profile by anatomic location in the normal population resembled that of pregnant women with fractures of the tibia and ankle, and forearm being the most common (incidences 150.8 and 120.8 per 100,000 person-years respectively). It was in fractures of the thigh where the largest proportional decrease was seen, where the fracture rate of the normal population was 3.6-fold. The smallest difference was seen in fractures of the pelvis where the fracture rate of the normal population was 1.8-fold compared to the pregnant population.

### Critical and multiple major fractures during pregnancy

A total of 39 patients had critical fractures concerning pregnancy with an incidence of 4.6/100,000 pregnancy-years (95% CI: 3–6). Rate of surgery was 21% (*n* = 8/39). All incidences remained markedly lower than in the normal population where the total incidence was 30.5 per 100,000 person-years. The incidence for multiple major fractures of the torso or proximal limbs was 1.6/100,000 pregnancy-years (95% CI: 1–3), and the rate of surgery was 9% (*n* = 1/11). Preterm delivery occurred in 21% (*n* = 8/39) and stillbirth in 10% (*n* = 4/39) of critical fractures. The most common fracture was a lumbar spinal column or sacral fracture (*n* = 7, incidence 0.8/100,000 pregnancy-years), followed by multiple spinal and pelvic fractures (*n* = 6, incidence 0.7/100,000 pregnancy-years), other fractures of the pelvis (*n* = 5, incidence 0.6/100,000 pregnancy-years) and fractures of the femoral neck (*n* = 4, incidence 0.5/100,000 pregnancy-years). It was specifically in fractures of the lumbar spine, the most common single fracture in pregnant women, the most dramatic decrease was seen with incidences remaining over tenfold smaller when compared to the normal population. Critical fractures resulted in preterm delivery in 14% of cases (*n* = 10/82). Subtrochanteric fractures (*n* = 1/2) and femoral shaft fractures (*n* = 1/2) had a rate of surgery of 50%. Sacral fractures had a rate of surgery of 29% (*n* = 2/7) and a preterm birth rate of 14% (*n* = 1/7). The stillbirth rate was 0% (*n* = 0/11). In cases of multiple pelvic fractures, the rate of surgery was 33% (*n* = 2/6); the preterm birth rate was 33% (*n* = 2/6), and the stillbirth rate was 17% (*n* = 1/6). Most of lumbar spine fractures were mostly treated conservatively (rate of surgery 14%, *n* = 1/7). These fractures resulted in preterm birth in 29% of cases (*n* = 2/7), and the stillbirth rate was 14% (*n* = 1/7). In total, lumbosacral and comminuted spinopelvic fractures resulted in preterm delivery in 25% (*n* = 5/20) of parturients, with a stillbirth rate of 10% (*n* = 2/20). Isolated fractures of the acetabulum and pubis were all treated conservatively until delivery, with no preterm births or stillbirths. The results are presented in more detail in Table 2.

**Table 2:** Incidences per 100,000 pregnancy-years of major fractures of the spinopelvic area divided by anatomical location (ICD-10 code) and multiple major fractures of the torso

Anatomical location	Count	Incidence (95% CI, surgical treatment %)		Preterm delivery (%)	Stillbirth (%)	Norma population incidence (95% CI, surgical treatment %)	
Fracture of lumbar vertebra (S32.0)	7	0.83	(0.33–1.69, 14%)	29	14	10.49	(8.17–13.26, 9%)
Fracture of sacrum (S32.1)	7	0.83	(0.33–1.69, 29%)	14	0	2.86	(1.73–4.46, 7%)
Fracture of acetabulum, pubis and ilium (S32.3, S32.4, S32.5)	6	0.71	(0.25–1.53, 0%)	0	0	4.67	(2.37–8.45, 10%)
Multiple fractures of lumbar spine and pelvis (S32.7)	6	0.71	(0.25–1.53, 33%)	33	17	2.78	(1.67–4.44, 20%)
Fracture of other parts of pelvis (S32.8)	5	0.59	(0.19–1.37, 0%)	0	0	2.12	(1.17–3.54, 5%)
Fracture of neck of femur (S72.0)	4	0.47	(0.13–1.12, 25%)	25	0	2.78	(1.67–4.36, 29%)
Per-, and subtrochanteric femur fractures and femoral shaft fractures (S72.1, S72.2, S72.3)	4	0.47	(0.13–1.12, 50%)	50	50	4.82	(2.6–8.52, 32%)
Total	39	4.6	(3.27–6.28, 21%)	21	10	30.52	(26.47–35.03, 16%)
Multiple major orthopedic fractures	14	1.65	(0.91–2.77, 7%)	14	7	24.27	(20.66–28.33, 36%)

Poisson exact-test was used to calculate 95% confidence intervals for incidences in brackets. Rates of surgical treatment, preterm delivery, and fetal mortality are shown as a percentage of the total count

## Discussion

In our nationwide study, the total incidence of fractures leading to hospitalization during pregnancy in the Finnish general population was 247 fractures per 100,000 pregnancy-years between 1998 and 2017. Approximately one quarter of these fractures required operative treatment, yielding a cumulative incidence of 61 operations per 100,000 pregnancy-years. With a Finnish population of 5.5 million, this amounts to a mean 27 operations yearly nationwide. During the study period, the yearly incidences of fractures and operations remained steady. Moreover, fractures of the tibia, ankle, and forearm made up half of all fractures and 65% of all operations. All fracture rates remained markedly lower than the age-adjusted female normal population. Maternal in-hospital mortality was low.

As previous nationwide studies on the incidence of fractures and fracture surgery on expectant mothers are non-existent, we are unable to compare our results to the findings of earlier studies. However, overall fracture epidemiology has been largely studied and trauma has been estimated to complicate one in 12 pregnancies [24]. Court-Brown et al. in Scotland in 2000 and Bergh et al. in Sweden between 2015 and 2018 reported whole-population fracture incidences for women of 1065 and 1413/100,000 person-years, respectively, incorporating both emergency department visits and hospitalizations [25, 26]. While these incidence figures are not necessarily comparable, all studies demonstrated that female fracture incidence remained fairly steady until onset of menopause, after which incidence started to grow rapidly.

In all the studied categories, our fracture incidence remained lower than the fracture incidence in the general population [12, 25, 27–29]. Fractures follow a different age distribution for men and women. For men, the distribution resembles a parabola with bias in the younger and older populations. In women, however, the incidence of fractures remains steady until a rapid increase occurs after menopause [25, 26]. This is due to the increase of osteoporotic fractures in the older population, which is exacerbated by bone demineralization in postmenopausal women [30]. The low total incidence of fractures in our study could in part be explained by our decision to include only pregnant women aged 15–49, and thereby excluded most osteoporotic fractures from the study. Lower incidences of hospitalized fractures can also be partly explained by the lower risk-taking behavior among pregnant women. This could be supported by the fact that the most dramatic decrease in incidence was seem in fractures of the thigh, which in the working population are traditionally high-energy fractures [31]. Furthermore, expectant women are often relieved from physically and mentally fatiguing work during early pregnancy and completely from all work during the third trimester, thus possibly making them less susceptible to work-related trauma. Pregnant women are advised against physical activity involving body contact or sudden movements and have been shown to spend more than half their time in a sedentary position [32, 33]. As a possible contributing factor, pregnant women are also encouraged to abstain from alcohol, with alcohol intoxication having been previously defined as a risk factor for traumatic events [34]. Lower incidence figures of hospitalized fractures also partially question the clinical importance of transient pregnancy-induced osteoporosis-related fractures during pregnancy. Transient osteoporosis has been shown to recover slowly after delivery, and these fractures occur and are mostly diagnosed post-partum after radiological and behavioral restrictions have been lifted [35–39].

Surgical rates were in line with previously defined whole-population values [12, 25, 27–29]. However, surgical rates for pelvic fractures were lower both when compared to the normal population in our study and previously defined figures for high-energy pelvic fractures, which have previously been shown to be predominant in the young [40, 41]. This finding suggests that surgeons are more cautious about operating on pelvic fractures in pregnant women when compared to the normal population. Pelvic fractures have been associated with adverse fetal and maternal outcomes, and a previous study by Weiss et al. defined the rate of fetal death due to maternal trauma at 3.7/100,000 live births [5, 42]. While the total stillbirth rate of 0.9/100,000 pregnancies (0.6%) for all fractures falls below previously defined figures, the stillbirth rate of 10% for comminuted spinopelvic and lumbosacral fractures remains relatively high.

The main strength of our study is the excellent national coverage of hospitalized fractures. Combined with the excellent national coverage of the Birth Register, we were able to create unique national data on hospitalized fractures during pregnancy and the subsequent delivery outcomes [15–17]. Thus, our results are provided good external validity. A secondary strength of our study is the long follow-up time (20 years). As a potential limitation, the Finnish national Birth Register only includes the outcomes of pregnancies with a viable fetus (≥ 22 weeks). Hence, fractures with termination of pregnancy before 22 full weeks were not included in our study. However, in pregnancies < 22 weeks, the fetus is enclosed in the womb and surrounded by soft tissue, where it is more protected. A second limitation of the study is that the Care Register only includes hospitalized fractures. Non-operatively treated minor fractures in Finland, such as fractures of the extremities, are primarily treated in a primary health care setting and are therefore not accurately registered in the Care Register. As a third limitation, we were only able to look at 39 critical fractures relating to pregnancy. This is attributed to the rare nature if these fractures when considering our long follow-up time and national coverage.

## Conclusion

Our results suggest that fractures during pregnancy occur more seldom than in the general fertile-aged female population, while their incidence has remained stable during the past 20 years. Interestingly, our results show that surgical rates remain lower than in the general female population, with especially lumbosacral and comminuted pelvic fractures having higher rates for preterm delivery. Despite this, maternal and fetal outcomes remained good. Operation rates varied on a yearly basis, suggesting that pregnant women with fractures are indeed dealt with delicately. We hope our results will serve as a basis for future studies and provide important epidemiological benchmark results of fractures during pregnancy.

## Supplementary Information

Below is the link to the electronic supplementary material.

Supplementary file1 (DOCX 23 KB)
